# Plasmon-Polariton Properties in Metallic Nanosphere Chains

**DOI:** 10.3390/ma8073910

**Published:** 2015-06-29

**Authors:** Witold Aleksander Jacak, Jurij Krasnyj, Andrej Chepok

**Affiliations:** 1 Department of Quantum Technology, Wrocław University of Technology, Wyb. Wyspiańskiego 27, Wrocław 50-370, Poland; 2 Department of Natural Science, Odessa Military Academy, Fontanskaya Doroga 10, Odessa 65009, Ukraine; E-Mail: peterkrasny@ukr.net; 3 IT Department, Odessa National Academy of Telecommunications (ONAT), 1 Kovalska str., Odessa 65029, Ukraine; E-Mail: andrew.chepok@gmail.com

**Keywords:** plasmons, metallic nano-chain, Lorentz friction, plasmon-polariton, radiative undamped propagation

## Abstract

The propagation of collective wave type plasmonic excitations along infinite chains of metallic nanospheres has been analyzed, including near-, medium- and far-field contributions to the plasmon dipole interaction with all retardation effects taken into account. It is proven that there exist weakly-damped self-modes of plasmon-polaritons in the chain for which the propagation range is limited by relatively small Ohmic losses only. In this regime, the Lorentz friction irradiation losses on each nanosphere in the chain are ideally compensated by the energy income from the rest of the chain. The completely undamped collective waves were identified in the case of the presence of persistent external excitation of some fragment of the chain. The obtained characteristics of these excitations fit the experimental observations well.

## 1. Introduction

The experimental and theoretical investigation of plasmon oscillations in metallic nanoparticles, besides its fundamental character, has also a great significance for applications. Metallic surface modifications at the nano-scale of solar cells exhibit a growth of the photo-voltaic efficiency due to the plasmon mediation in sun light energy harvesting [1–6]. On the other hand, periodic linear structures of metallic nanoparticles serve as plasmon wave-guides with low damping [7–9]. The wave-lengths propagating in such structures’ plasmon-polaritons are considerably shorter in comparison to light with the same frequency [10–12]. This allows for avoiding the diffraction limits for light circuits when one transforms the light signal into the plasmon-polariton wave [13–15]. This is treated as prospective for the forthcoming construction of plasmon opto-electronic nano-devices that are not available in ordinary light wave-guides, because of diffraction constraints.

Metallic clusters of a size of 1–2 nm were widely investigated in the 1980s of the last century, both theoretically within the so-called time-dependent local density approximation (TDLDA) [16–19], and experimentally, especially for small clusters of alkali metals, like Na or K. Several unique properties of such systems were recognized and described, as, e.g., the so-called spill-out of electron liquid beyond the rim of an ion lattice and the influence of this phenomenon on plasmon oscillations in small clusters. Nevertheless, later, for analyzing larger metallic nanoparticles of a size of several tens of nm, other phenomena were indicated, in particular linked to their pronounced radiative properties overwhelming the plasmon behavior in this larger size scale. To describe plasmon properties in relatively large electron nano-systems, with *∼* 10^5^*^−^*^7^ electrons, the numerically accurate methods based on the Kohn–Sham approach (TDLDA) are not useful, because of numerical efficiency constraints (these methods are limited rather to much smaller numbers of electrons, 200–300 as in the case of the 1–2 nm scale clusters). For larger nanoparticles with a size of tens of nanometers, various variants of random phase approximation (RPA) turned out to be appropriate and useful [16,17,20]. Especially, much attention is focused on large nanoparticles of noble metals (gold, silver and copper) because of the location of surface plasmon resonances in particles of these metals within the visible light spectrum.

Due to intensive irradiation of surface plasmons in large nanoparticles, special interest has been paid to plasmon-polariton wave-guides in the form of chains consisting of these nanoparticles [21–23]. Near-field microscopy applied to such systems directly demonstrated long-range propagation of the plasmon-polariton signal along these structures. For instance, in [24], the observation of a 5 µm-range propagation in gold nanoparticles with an average radius *a* = 50 nm aligned in the equidistant chain with separation between neighboring spheres *d* = 200 nm has been reported. In a series of papers [10,25–28], practically non-damped propagation of collective plasmonic modes in gold and silver nano-chains has been demonstrated over distances of *∼* 0.5 µm, and the energy transfer along the chain was directly confirmed. Such a long range of plasmon-polariton propagation reaching visible light wave-lengths and undergoing reduced group velocity below 0.1*×c* [10,26,28] fit well with the requirements of subdiffraction nano-photonics. In [29], the weakly-damped propagation of plasmon-polaritons along silver nanowires with diameters of *ca*. *a* = 100 nm over distances of *ca*. 20 µm has also been reported. In [11], the similarly long range for the practically undamped propagation of plasmon-polaritons in various metallic nanostructures has been summarized. The theoretical estimation of plasmon-polariton attenuation in metallic nano-chains made with a dipole-dipole interaction model using finite elements FDDTnumerical methods leads to rather larger attenuation (*ca*. 3 dB/15 µm) than that observed experimentally [11,24].

In the present paper, we try to resolve the problem of this discrepancy within the rigorous treatment of collective surface plasmon propagation along the nano-chain, including retardation effects for the near-, medium- and far-field zone of plasmon dipoles and describing plasmon oscillations in individual nanospheres with the RPA approach. In the present paper, we analyze the surface dipole-type plasmons in a single nanosphere with the formerly formulated RPA model [30,31], including damping effects, in particular of the Lorentz friction type for plasmons. Next, the radiative properties of collective surface plasmons in nanospheres located in the chain of metallic nanospheres are studied and described taking into account the near-, medium- and far-field contributions to dipole coupling between elements of the chain and including the retardation effects. The ideal vanishing of radiation losses in the chain for a wide spectrum of collective plasmonic wave-type modes is demonstrated. The undamped plasmon-polariton modes induced by the external persistent oscillating electric forcing field were identified. The frequency of these modes equals the frequency of the external field, and their amplitude is maximal for the resonance with plasmon-polariton self-mode frequencies. The calculus of plasmon self-mode frequencies, their attenuation rates and group velocities are presented for longitudinal and transverse polarizations of collective excitations and for the wide range of chain parameters. A problem of the logarithmic singular contribution caused by the far-field component of the dipole interaction has also been resolved, for the self-energy of plasmon-polaritons with transverse polarization and the similar one caused by the medium-field component for the group velocity of both polarizations, with some link to a recent discussion of numerical simulations of long-range kinetics of plasmon-polaritons in metallic nano-chains [32–36].

## 2. Plasmon Oscillations in Metallic Nanospheres Induced by an Electric Field Signal with the Inclusion of Plasmon Damping

We will consider a chain of metallic (Au or Ag) nanospheres of the radius *a* located in a dielectric environment with the dielectric constant *ε* in the presence of the dynamic external electric field applied to some part of the chain. We will analyze a collective reaction to this field of the electron liquid in metallic components of the chain. To describe this composite system, let us first analyze plasmon oscillations in the single metallic nanosphere with special attention paid to surface plasmon damping caused by the irradiation of the electromagnetic wave by electron plasma oscillations.

We assume the jellium model for each metallic nanosphere in which the positive charge of ion background is static and uniformly smeared over the whole nanosphere, *n_e_*(*r*) = *n_e_*Θ(*a − r*), *n_e_* = *N_e_/V*, where *N_e_* is the number of free electrons in the nanosphere; V is the volume of the nanosphere; Θ is the Heaviside step function; |*e|n_e_* is the averaged density of the positive charge compensating the charge of free electrons.

Fluctuations of electron local density in the nanosphere can be divided into surface and volume parts [30],

(1) $$\delta \rho (r,t)=\{\begin{array}{l} \delta \rho_{1}(r,t),\;for\;r<a\\ \delta \rho_{2}(r,t),\;for\;r\geq a,(r\to a+) \end{array}$$

These two parts of local electron density fluctuations satisfy the equations derived in the framework of random phase approximation (RPA) [30]:

(2) $$\frac{\partial^{2}\delta \rho_{1}(r,t)}{\partial t^{2}}=\frac{2}{30}\frac{\epsilon_{F}}{m}\nabla^{2}\delta \rho_{1}(r,t)-\omega_{p}^{2}\delta \rho_{1}(r,t)$$

and:

(3) $$\begin{array}{l} \frac{\partial^{2}\delta \rho_{2}(r,t)}{\partial t^{2}}=-\frac{2}{3m}\nabla \left\{\left[\frac{2}{5}\epsilon_{F}n_{e}+\epsilon_{F}\delta \rho_{2}(r,t)\right]\frac{r}{r}\delta (r-a)\right\}\\ -[\frac{2}{3}\frac{\epsilon_{F}}{m}\frac{r}{r}\nabla \delta \rho_{2}(r,t)+\frac{\omega_{p}^{2}}{4\pi }\frac{r}{r}\nabla \int d^{3}r_{1}\frac{1}{\left|r-r_{1}\right|}\\ \times (\delta \rho_{1}(r_{1},t)\Theta (a-r_{1})+\delta \rho_{2}(r_{1},t)\Theta (r_{1}-a))]\delta (r-a), \end{array}$$

where $\omega_{p}^{2}=\frac{4\pi n_{e}e^{2}}{m}$ is the bulk plasmon frequency; *ϵ_F_* is the Fermi energy; *n_e_* is the equilibrium density of electrons (equal to jellium density) and *m* is the electron mass. The analysis and solution of the above equations have been done in [30], resulting in the determination of the plasmon self-modes spectrum, both for volume and surface modes.

Nevertheless, this RPA treatment did not account for the plasmon damping. One can, however, include the damping of plasmons in a phenomenological manner, by adding an attenuation term to plasmon dynamic oscillatory-type equations, *i.e.*, by adding the terms $-\frac{2}{\tau_{0}}\frac{\partial \delta \rho (r,t)}{\partial t}$ to the r.h.s. of both Equations (2) and (3) [30]. The damping rate $\frac{1}{\tau_{0}}$ accounts for electron scattering losses [25],

(4) $$\frac{1}{\tau_{0}}≃\frac{v_{F}}{2\lambda_{b}}+\frac{Cv_{F}}{2a},$$

where *C* is the constant of unity order; *a* is the nano-sphere radius; *v_F_* is the Fermi velocity in the metal; *λ_b_* is the electron mean free path in bulk metal (including scattering of electrons on other electrons, on impurities and on phonons [25]); e.g., for Au, *v_F_* = 1.396 *×* 10^6^ m/s and λ*_b_* ≃ 53 nm (at room temperature); the latter term in Equation (4) accounts for scattering of electrons on the boundary of the nanoparticle, while the former one corresponds to scattering processes similar as in the bulk. The other effects, such as the so-called Landau damping (especially important in small clusters [19,37]), corresponding to the decay of a plasmon for a high-energy particle-hole pair, are of lessening significance for nano-sphere radii larger than 2–3 nm [37] and are completely negligible for radii larger than 5 nm (we will consider here the large nanospheres with radii *≥* 10 nm). Note that a similarly lessening role as with the radius growth is played also by the electron liquid spill-out effect [16,18], though this was of primary importance for small clusters [16,20].

Besides homogeneous Equations (2) and (3) determining the self-frequencies of plasmon modes, the dual inhomogeneous equations would be written, with an explicit expression for the forcing factor. This factor would be the time-dependent electric field, e.g., the electric component of the incident e-mwave. For the e-m wave frequency that is in resonance with plasmons in the metallic nanosphere, the wave-length (being of order of 500 nm in this case) highly exceeds the nanosphere size (with radius 10–50 nm), thus the dipole regime conditions are fulfilled. For the forcing field **E**(*t*), almost homogeneous over the nanosphere (which corresponds to the dipole approximation), only the dipole surface mode can be excited, and the electron liquid response resolves to a single dipole-type mode, described by the function *Q*_1_*_m_*(*t*) (*i.e.*, with *l* = 1 and *m* = −1, 0, 1, the angular momentum numbers related to spherical symmetry and describing the dipole mode). The function *Q*_1_*_m_*(*t*) satisfies the equation:

(5) $$\begin{array}{l} \frac{\partial^{2}Q_{1m}(t)}{\partial t^{2}}+\frac{2}{\tau_{0}}\frac{\partial Q_{1m}(t)}{\partial t}+\omega_{1}^{2}Q_{1m}(t)\\ =\sqrt{\frac{4\pi }{3}}\frac{en_{e}}{m}[E_{z}(t)\delta_{m,0}+\sqrt{2}(E_{x}(t)\delta_{m,1}+E_{y}(t)\delta_{m,-1})], \end{array}$$

where $\omega_{1}=\frac{\omega_{p}}{\sqrt{3\varepsilon }}$ (this is a dipole surface plasmon Mie-type frequency [38], and *ε* is the dielectric susceptibility of the nanosphere surroundings [30]). Only this function contributes to the plasmon response to the homogeneous electric field. Thus, for the homogeneous forcing field, the electron density fluctuations attain the form [30]:

(6) $$\delta \rho (r,t)=\left\{ \begin{array}{l} 0,\;r<a\\ \sum_{m=-1}^{1}Q_{1m}(t)Y_{1m}(\Omega ),\;r\geq a\;r\to a+ \end{array}\right.$$

where *Y_lm_*(Ω) is the spherical function with *l* = 1.

For plasmon oscillations given by Equation (6), one can calculate the corresponding dipole **D**(*t*),

(7) $$\{\begin{array}{c} D_{x}(t)=e\int d^{3}rx\delta \rho (r,t)=\frac{\sqrt{2\pi }}{\sqrt{3}}eQ_{1,1}(t)a^{3}\\ D_{y}(t)=e\int d^{3}ry\delta \rho (r,t)=\frac{\sqrt{2\pi }}{\sqrt{3}}eQ_{1,-1}(t)a^{3}\\ D_{z}(t)=e\int d^{3}rz\delta \rho (r,t)=\frac{\sqrt{4\pi }}{\sqrt{3}}eQ_{1,0}(t)a^{3} \end{array}$$

and **D**(*t*) satisfies the equation (rewritten Equation (5)),

(8) $$\left[\frac{\partial^{2}}{\partial t^{2}}+\frac{2}{\tau_{0}}\frac{\partial }{\partial t}+\omega_{1}^{2}\right]D(t)=\frac{a^{3}4\pi e^{2}n_{e}}{3m}E(t)=\varepsilon a^{3}\omega_{1}^{2}E(t).$$

The phenomenological oscillatory-type damping term in the above equation includes all types of scattering phenomena, *i.e.*, electron-electron, electron-phonon, electron-admixture interactions, as well as the contribution caused by the boundary scattering effect [25]. Electron scattering effects cause significant attenuation of plasmons, especially important in small metal clusters. These contributions to the damping time rate are proportional to $\frac{1}{a}$ and are of lessening significance with the radius growth. There is, however, also an important channel of plasmon damping caused by radiation losses, not included in the formula for *τ*_0_. We will show that the contribution to the overall attenuation of plasmons caused by the radiation losses scales as *a*^3^, and therefore, for large nano-spheres, the radiative losses dominate the plasmon damping.

The radiative losses of oscillating charges related to plasmon dipole variation in time can be expressed by the Lorentz friction [39], *i.e.*, by the equivalent fictitious electric field slowing down the motion of charges,

(9) $$E_{L}=\frac{2}{3c^{3}}\frac{\partial^{3}D(t)}{\partial t^{3}}.$$

Thus, one can rewrite Equation (8) including the Lorentz friction term,

(10) $$\left[\frac{\partial^{2}}{\partial t^{2}}+\frac{2}{\tau_{0}}\frac{\partial }{\partial t}+\omega_{1}^{2}\right]D(t)=\varepsilon a^{3}\omega_{1}^{2}E(t)+\varepsilon a^{3}\omega_{1}^{2}E_{L},$$

or more explicitly, for the case when **E** = 0:

(11) $$\left[\frac{\partial^{2}}{\partial t^{2}}+\omega_{1}^{2}\right]D(t)=\frac{\partial }{\partial t}\left[\frac{2}{\tau_{0}}D(t)+\frac{2}{3\omega_{1}\sqrt{\varepsilon }}{\left(\frac{\omega_{p}a}{c\sqrt{3}}\right)}^{3}\frac{\partial^{2}}{\partial t^{2}}D(t)\right].$$

Applying now the perturbation procedure for solving Equation (11) and treating the r.h.s of this equation as the perturbation, one obtains in the zeroth step of perturbation $\left[\frac{\partial^{2}}{\partial t^{2}}+\omega_{1}^{2}\right]D(t)=0$, from which $\frac{\partial^{2}}{\partial t^{2}}D(t)=-\omega_{1}^{2}D(t)$. Therefore, with the first step of perturbation, one can substitute the latter formula to the r.h.s. of Equation (11), *i.e.*,

(12) $$\left[\frac{\partial^{2}}{\partial t^{2}}+\frac{2}{\tau }\frac{\partial }{\partial t}+\omega_{1}^{2}\right]D(t)=0,$$

where

(13) $$\frac{1}{\tau }=\frac{1}{\tau_{0}}+\frac{\omega_{1}}{3\sqrt{\varepsilon }}{\left(\frac{\omega_{p}a}{c\sqrt{3}}\right)}^{3}.$$

In this way, we have included the Lorentz friction into the total attenuation rate $\frac{1}{\tau }$, which is justified for non-extremely large nanospheres, *i.e.*, when the second term in Equation (13), proportional to *a*^3^, is sufficiently small to fulfill the perturbation procedure constraints. For nanospheres with a radius of 10–30 nm, this approximation is justified and was verified experimentally for Au and Ag nanospheres [31]. It is worth emphasizing the ability of this approach to explain an experimentally-observed redshift of the resonance of the surface plasmon frequency with growing *a* (visible in the extinction spectrum of light passing the water colloidal solutions of metallic nanospheres with various radii [31]). Indeed, the solution of Equation (12) is of the form $D(t)=Ae^{-t/\tau }\cos({\omega^{\prime }}_{1}t+\phi )$, where ${\omega^{\prime }}_{1}={\omega^{\prime }}_{1}\sqrt{1-\frac{1}{{\left(\omega_{1}\tau \right)}^{2}}}$, which gives the experimentally-observed red shift of the plasmon resonance due to ∼ *a*^3^ growth of the attenuation caused by the irradiation losses. The Lorentz friction term in Equation (13) dominates plasmon damping for *a ≥* 12 nm (for Au and Ag) due to this *a*^3^ dependence. The plasmon damping grows rapidly with *a* and results in pronounced redshift of resonance frequency in good coincidence with the experimental data for 10 < *a* < 30 nm (Au and Ag) [31]. In this region for the size of metallic particles, we deal with conspicuous cross-over of the attenuation rate in its dependence on *a*. The minimum of damping is achieved at:

(14) $$a^{*}={\left(\frac{9}{2}\varepsilon \frac{v_{F}c^{3}}{\omega_{p}^{4}}\right)}^{1/4}$$

and for a <a^*^, the damping rate grows with lowering *a* approximately as $\sim \frac{1}{a}$, while for *a > a^∗^*, this rate grows as *a*^3^ with rising *a*. The value of *a^∗^* can be estimated for Au, Ag and Cu; *cf*. Table 1.

## 3. Radiative Properties of the Metallic Nanosphere in the Chain

When a metallic nanosphere is an element of the chain created by similar nanospheres equidistantly-distributed along a line, one has to take into account that its irradiation losses may be modified by energy income from other nanospheres when in the total system, the collective plasmon excitation propagates. The periodicity of the chain makes the system similar to a 1D crystal. The interaction between nanospheres can be considered as of the dipole-type coupling. The minimal separation of nanospheres in the chain is *d* = 2*a* (*d* is the distance between neighboring sphere centers), but we will consider *d* > 3*a*, because the dipole approximation of plasmon interaction in the nanosphere-chain is sufficiently accurate for *d* > 3*a*, when the contribution of the multipole interaction can be neglected. Various numerical large-scale calculations of the e-m field distribution in such systems were done, including dipole and also multipole interaction between plasmonic oscillations in metallic components [21–23]. It is worth noting that the model of interacting dipoles [40,41] was developed earlier for the investigation of stellar matter [42,43], and next, it was adopted for metal particle systems [44,45]. The numerical studies beyond the dipole model [21,23] indicated that the dipole model is sufficiently accurate when the particle separation is not lower than the particle dimensions. Otherwise, the multipole contribution to the interaction starts to be important [46].

Assuming that in the sphere located in the point **r**, we deal with the dipole **D**, then in the other place **r**_0_ (**r**_0_ is fixed to the end of **r**), this dipole causes an electric field in the form as follows (including the relativistic retardation of electromagnetic signals) [39,47]:

(15) $$\begin{array}{l} E(r,r_{0},t)=\frac{1}{\varepsilon }\left(-\frac{\partial^{2}}{v^{2}\partial t^{2}}\frac{1}{r_{0}}-\frac{\partial }{v\partial t}\frac{1}{r_{0}^{2}}-\frac{1}{r_{0}^{3}}\right)D(r,t-r_{0}/v)\\ +\frac{1}{\varepsilon }\left(\frac{\partial^{2}}{v^{2}\partial t^{2}}\frac{1}{r_{0}}+\frac{\partial }{v\partial t}\frac{3}{r_{0}^{2}}+\frac{3}{r_{0}^{3}}\right)n_{0}(n_{0}\cdot D(rt-t_{0}/v)), \end{array}$$

where $n_{0}=\frac{r_{0}}{r_{0}}$ and $v=\frac{c}{\sqrt{\epsilon }}$. The above formula includes the terms corresponding to the near-field zone (denominator with $r_{0}^{3}$), medium-field zone (denominator with $r_{0}^{2}$ and far-field zone (denominator with *r*_0_) contributions to the dipole field. This allows for writing out the dynamical equation for plasmon oscillations at each nanosphere of the chain, which can be numbered by the integer *l* (*d* denotes the separation between nanospheres in the chain, *d* > 3*a*; the vectors **r** and **r**_0_ are collinear if the origin is associated with one of the nanospheres in the chain).

Therefore, the equation for the surface plasmon oscillations on the *l*-th sphere is as follows,

(16) $$\begin{array}{l} \left[\frac{\partial^{2}}{\partial t^{2}}+\frac{2}{\tau_{0}}\frac{\partial }{\partial t}+\omega_{1}^{2}\right]D_{\alpha }(ld,t)\\ =\varepsilon \omega_{1}^{2}a^{3}\sum_{m=-\infty ,m\neq l}^{m=\infty }E_{\alpha }\left(md,t-\frac{|l-m|d}{c}\right)+\varepsilon \omega_{1}^{2}a^{3}E_{L\alpha }(ld,t)+\varepsilon \omega_{1}^{2}a^{3}E_{\alpha }(ld,t). \end{array}$$

The first term of the r.h.s. in Equation (16) describes the dipole-type coupling between nanospheres, and the other two terms correspond to the contribution to plasmon attenuation due to the Lorentz friction (as described in the previous section) and the forcing field due to an external electric field. The index *α* enumerates polarizations, longitudinal *α* = *z* and transverse *α* = *x*(*y*), with respect to the chain orientation (assumed in the *z* direction). According to Equation (15), we have:

(17) $$\begin{array}{l} E_{z}(md,t)=\frac{2}{\varepsilon d^{3}}\left(\frac{1}{{\left|m-l\right|}^{3}}+\frac{d}{v{\left|m-l\right|}^{2}}\frac{\partial }{\partial t}\right)D_{z}(md,t-\left|m-l\right|d/v)\\ E_{x(y)}(md,t)=-\frac{1}{\varepsilon d^{3}}\left(\frac{1}{{\left|m-l\right|}^{3}}+\frac{d}{v{\left|m-l\right|}^{2}}\frac{\partial }{\partial t}+\frac{d^{2}}{v^{2}\left|l-d\right|}\frac{\partial^{2}}{\partial t^{2}}\right)D_{x(y)}(md,t-\left|m-l\right|d/v). \end{array}$$

Taking advantage of the chain periodicity (in analogy to the Bloch states in crystals with the reciprocal lattice of quasi-momentum), one can assume,

(18) $$\begin{array}{l} R_{\alpha }(ld,t)={\tilde{R}}_{\alpha }(k,t)e^{-ikld}\\ 0\leq k\leq \frac{2\pi }{d}. \end{array}$$

One can assert it in a more formal manner taking the Fourier picture of Equation (16). As dipoles are localized on nanospheres in their centers, the system is discrete, similar to the case of phonons in a 1D crystal. One can thus apply the discrete Fourier transform (DFT) with respect to the positions, while the ordinary continuous Fourier transform (CFT) with respect to time. DFT is defined for a finite set of numbers, so we can consider the chain with 2*N* + 1 nanospheres, *i.e.*, the chain of length *L* = 2*Nd*. Thus, for any discrete characteristics *f*(*l*), *l* = −*N*, …, 0, …, *N* of the chain, like a selected polarization of dipole distribution, one deals with the DFT picture $f(k)=\sum_{l=-N}^{N}f(l)e^{ikld}$, where $k=\frac{2\pi }{2Nd}n$, *n* = 0, …, 2*N*. This means that *kd* ∈ [0, 2*π*] due to the periodicity of the equidistant chain with the separation between nanosphere centers equal to *d*. On the whole system, the Born–Karman boundary condition is imposed, resulting in the above form of *k*. In order to account for the infinite length of the chain, one can take the limit *N → ∞*, which causes that the variable *k* is quasi-continuous, but still *kd* ∈ [0, 2*π*].

As is described in Section 4, one arrives at the Fourier picture of Equation (16), DFT for positions and CFT for time,

(19) $$\begin{array}{l} \left(-\omega^{2}-i\frac{2}{\tau_{0}}\omega +\omega_{1}^{2}\right)D_{\alpha }(k,\omega )\\ =\omega_{1}^{2}\frac{a^{3}}{d^{3}}F_{\alpha }(k,\omega )D_{\alpha }(k,\omega )+\varepsilon a^{3}\omega_{1}^{2}E_{0\alpha }(k,\omega ), \end{array}$$

with:

(20) $$\begin{array}{l} F_{z}(k,\omega )=4\sum_{m=1}^{\infty }\left(\frac{\cos(mkd)}{m^{3}}\cos(m\omega d/v)+\omega d/v\frac{\cos(mkd)}{m^{2}}\sin(m\omega d/v)\right)\\ +2i[\frac{1}{3}{\left(\omega d/v\right)}^{3}+2\sum_{m=1}^{\infty }(\frac{\cos(mkd)}{m^{3}}\sin(m\omega d/v)\\ -\omega d/v\frac{\cos(mkd)}{m^{2}}\cos(m\omega d/v))],\\ F_{x(y)}(k,\omega )=-2\sum_{m=1}^{\infty }(\frac{\cos(mkd)}{m^{3}}\cos(m\omega d/v)+\omega d/v\frac{\cos(mkd)}{m^{2}}\sin(m\omega d/v)\\ -{\left(\omega d/v\right)}^{2}\frac{\cos(mkd)}{m}\cos(m\omega d/v))\\ -i[-\frac{2}{3}{\left(\omega d/v\right)}^{3}+2\sum_{m=1}^{\infty }(\frac{\cos(mkd)}{m^{3}}\sin(m\omega d/v)+\omega d/v\frac{\cos(mkd)}{m^{2}}\cos(m\omega d/v)\\ -{\left(\omega d/v\right)}^{2}\frac{\cos(mkd)}{m}\sin(m\omega d/v))]. \end{array}$$

**Figure 1.**
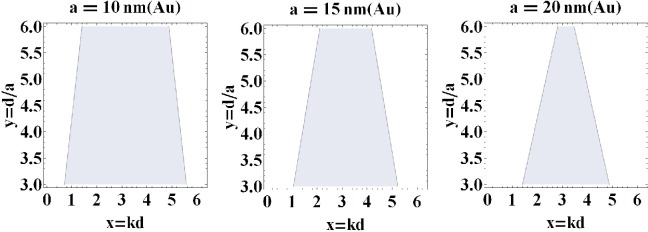
The region (marked as shadow, 0 < *kd ± ω*_1_*d/v* < 2*π*) in which the radiation losses vanish for infinite chains of Au nanospheres with radius *a* = 10, 15, 20 nm and chain-separation *d/a* ∈ [3, 6] (*ω* = *ω*_1_, *v* = *c*).

**Figure 2.**
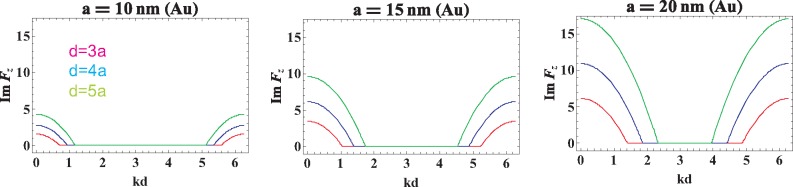
The function *ImF_z_*(*k*; *ω* = *ω*_1_) for infinite chains of Au nanospheres with radius *a* = 10, 15, 20 nm and chain-separation *d* = 3*a*, 4*a*, 5*a*.

**Figure 3.**
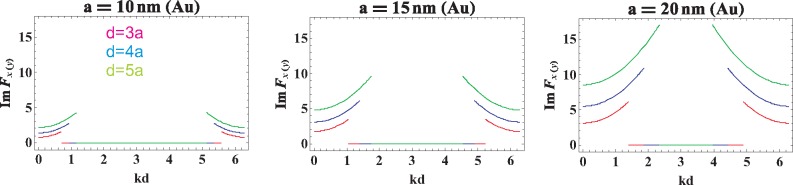
The function *ImF_x_*_(_*_y_*_)_(*k*; *ω* = *ω*_1_) for infinite chains of Au nanospheres with radius *a* = 10, 15, 20 nm and chain-separation *d* = 3*a*, 4*a*, 5*a*.

The direct calculation of the functions *ImF_z_*(*k, ω*) and *ImF_x_*_(_*_y_*_)_(*k*, **ω**), which correspond to the radiative damping for the longitudinal and transverse plasmon-polariton polarizations, respectively, is done in the Section 4, Equations (26) and (28). We have shown there that both of these functions vanish when 0 < *kd* ± **ω***d/v* < 0 (the corresponding region is indicated in Figure 1). Outside of this region, the radiative damping expressed by the functions *ImF_α_*(*k*, **ω**) is not zero, which for the longitudinal and transverse modes is illustrated in Figures 2 and 3, correspondingly.

## 4. Calculation of the Radiative Damping of the Plasmon-Polariton in the Chain

Both sides of Equation (16) can be multiplied by $\frac{e^{i}(kld-\omega t)}{2\pi }$, and next, one can perform summation with respect to the nanosphere positions and integration over *t*. Taking into account that,

(21) $$\begin{array}{l} \frac{1}{2\pi }\int_{\infty }^{\infty }\sum_{l=-N}^{N}D_{\alpha }(\pm md+ld;t-\frac{md}{v})e^{-i(kld-\omega t)}\\ =e^{i(\mp kmd+\omega \frac{md}{v})}D_{\alpha }(l,\omega ), \end{array}$$

one obtains thus the following equation in Fourier representation (the discrete Fourier transform for nanosphere positions and the continuous Fourier transforms for time),

(22) $$\begin{array}{l} \left(-\omega^{2}-i\frac{2}{\tau_{0}}\omega +\omega_{1}^{2}\right)D_{\alpha }\left(k,\omega \right)\\ =\omega_{1}^{2}\frac{\alpha^{3}}{d^{3}}F_{\alpha }\left(k,\omega \right)D_{\alpha }\left(k,\omega \right)+\varepsilon a^{3}\omega_{1}^{2}E_{0\alpha }\left(k,\omega \right), \end{array}$$

where $k=\frac{2\pi n}{2Nd}$, *n* = 0, 1,…, 2*N*, *i.e.*, *kd* ∈ [0, 2*π*], due to the periodicity of the chain with equidistant separation *d* of nanospheres, and the form of *k* is due to the Born–Karman boundary condition with the period *L* = 2*Nd*. For *N → ∞* (infinite chain limit), *k* is a quasi-continuous variable. In Equation (22),

(23) $$\begin{array}{l} F_{z}\left(k,\omega \right)=4\sum_{m=1}^{\infty }\left(\frac{cos\left(mkd\right)}{m^{3}}cos\left(m\omega d/v\right)+\omega d/v\frac{cos\left(mkd\right)}{m^{2}}sin\left(m\omega d/v\right)\right)\\ +2i[\frac{1}{3}{\left(\omega d/v\right)}^{3}+2\sum_{m=1}^{\infty }(\frac{cos(mkd)}{m^{3}}sin(m\omega d/v)\\ -\omega d/v\frac{cos(mkd)}{m^{2}}cos(m\omega d/v))],\\ F_{x(y)}\left(k,\omega \right)=-2\sum_{m=1}^{\infty }(\frac{cos(mkd)}{m^{3}}cos(m\omega d/v)+\omega d/v\frac{cos(mkd)}{m^{2}}sin(m\omega d/v)\\ -{\left(\omega d/v\right)}^{2}\frac{cos\left(mkd\right)}{m}cos\left(m\omega d/v\right)\\ -i[-\frac{2}{3}{\left(\omega d/v\right)}^{3}+2\sum_{m=1}^{\infty }(\frac{cos(mkd)}{m^{3}}sin(m\omega d/v)\omega d/v\frac{cos(mkd)}{m^{2}}cos(m\omega d/v)\\ -{\left(\omega d/v\right)}^{2}\frac{cos(mkd)}{m}sin(m\omega d/v))]. \end{array}$$

Some summations in the above equations can be done analytically [48]:

(24) $$\left\{ \begin{array}{l} \sum_{m=1}^{\infty }\frac{sin(mz)}{m}=\frac{\pi -z}{2},\;for\;0<z<2\pi \\ \sum_{m=1}^{\infty }\frac{cos(mz)}{m}=\frac{1}{2}ln\left(\frac{1}{2-2cos(z)}\right)\\ \sum_{m=1}^{\infty }\frac{cos(mz)}{m^{2}}=\frac{\pi^{2}}{6}-\frac{\pi }{2}z+\frac{1}{4}z^{2},\;for\;0<z<2\pi \\ \sum_{m=1}^{\infty }\frac{sin(mz)}{m^{3}}=\frac{\pi^{2}}{6}z-\frac{\pi }{2}z^{2}+\frac{1}{12}z^{3},\;for\;0<z<2\pi \end{array}\right.$$

Using the above formulae, one can show that if 0 < *kd ± ωd/v* < 2*π*, then:

(25) $$\begin{array}{l} ImF_{z}(k,\omega )=2\sum_{m=1}^{\infty }[\frac{sin(m(kd+\omega d/v))-sin(m(kd-\omega d/v))}{m^{3}}\\ -(\omega d/v)\frac{cos(m(kd+\omega d/v))+cos(m(kd-\omega d/v))}{m^{2}}]+\frac{2}{3}{\left(\omega d/v\right)}^{3}\\ =2\left[\frac{\pi^{2}}{6}(kd+\omega d/v)-\frac{\pi }{4}{\left(kd+\omega d/v\right)}^{2}+\frac{1}{12}{\left(kd+\omega d/v\right)}^{3}\right]\\ -2\left[\frac{\pi^{2}}{6}(kd-\omega d/v)-\frac{\pi }{4}{\left(kd+\omega d/v\right)}^{2}+\frac{1}{12}{\left(kd-\omega d/v\right)}^{3}\right]\\ -2(\omega d/v)\left[\frac{\pi^{2}}{6}-\frac{\pi }{2}(kd+\omega d/v)+\frac{1}{4}{\left(kd+\omega d/v\right)}^{2}\right]\\ -2(\omega d/v)\left[\frac{\pi^{2}}{6}-\frac{\pi }{2}(kd-\omega d/v)+\frac{1}{4}{\left(kd-\omega d/v\right)}^{2}\right]+\frac{2}{3}{\left(\omega d/v\right)}^{3}\equiv 0. \end{array}$$

However, if *kd−ωd/v* < 0 or *kd*+*ωd/v* > 2*π* for some values of wave vector *k*, then a more general formula must be used (by the utilization of the Heaviside step function, one can extend Equation (24) to the second period of their left sides; note that for *d/a* ∈ [2, 10], *a* < 25 nm, the next periods over the second one are not reached for *kd* ∈ [0, 2*π*)). This extended form for *ImF_z_*(*k, ω*) is as follows (here, we use dimensionless variables *x* = *kd, y* = *d/a*):

(26) $$\begin{array}{l} ImF_{z}(k,\omega )=\Theta (2\pi -x-\omega ya/v)2\left[\frac{\pi^{2}}{6}(x+\omega ya/v)-\frac{\pi }{4}{\left(x+\omega ya/v\right)}^{2}+\frac{1}{12}{\left(x+\omega ya/v\right)}^{3}\right]\\ \Theta (-2\pi +x+\omega ya/v)2\left[\frac{\pi^{2}}{6}(x+\omega ya/v-2\pi )-\frac{\pi }{4}{\left(x+\omega ya/v-2\pi \right)}^{2}+\frac{1}{12}{\left(x+\omega ya/v-2\pi \right)}^{3}\right]\\ -\Theta (x-\omega ya/v)2\left[\frac{\pi^{2}}{6}(x-\omega ya/v)-\frac{\pi }{4}{\left(x-\omega ya/v\right)}^{2}+\frac{1}{12}{\left(x-\omega ya/v\right)}^{3}\right]\\ -\Theta (-x+\omega ya/v)2\left[\frac{\pi^{2}}{6}(x-\omega ya/v+2\pi )-\frac{\pi }{4}{\left(x-\omega ya/v+2\pi \right)}^{2}+\frac{1}{12}{\left(x-\omega ya/v+2\pi \right)}^{3}\right]\\ -\Theta (2\pi -x-\omega ya/v)2(\omega ya/v)\left[\frac{\pi^{2}}{6}-\frac{\pi }{2}(x+\omega ya/v)+\frac{1}{4}{\left(x+\omega ya/v\right)}^{2}\right]\\ -\Theta (-2\pi +x+\omega ay/v)2(\omega ay/v)\left[\frac{\pi^{2}}{6}-\frac{\pi }{2}(x+\omega ya/v-2\pi )+\frac{1}{4}{\left(x+\omega ya/v-2\pi \right)}^{2}\right]\\ -\Theta (-x+\omega ay/v)2(\omega ay/v)\left[\frac{\pi^{2}}{6}-\frac{\pi }{2}(x-\omega ya/v+2\pi )+\frac{1}{4}{\left(x-\omega ya/v+2\pi \right)}^{2}\right]\\ -\Theta (x-\omega ay/v)2(\omega ay/v)\left[\frac{\pi^{2}}{6}-\frac{\pi }{2}(x-\omega ya/v)+\frac{1}{4}{\left(x-\omega ya/v\right)}^{2}\right]+\frac{2}{3}{\left(\omega ay/v\right)}^{3}. \end{array}$$

The function given by Equation (26) is depicted in Figure 2. Equation (26) allows one to account for the inconsistency of the periodic functions given by the sums of sines and cosines with the non-periodic Lorentz friction term and the inconsistency of arguments *kd*±**ω***d/v* of trigonometric functions out of the first period. In Figure 1, we have plotted the solution of the equation (*kd−*ω*d/v*)(*kd*+ω*d/v−*2*π*) = 0, which determines the region for *kd* (denoted by *x*) *versus d/a* (denoted by *y*) inside which the exact cancellation of the Lorentz friction by radiative energy income from other nanospheres takes place. In Figure 2, the comparison of this cancellation for various nanosphere diameters is presented, for the longitudinal polarization of plasmon collective excitations.

A similar analysis can be done for the transverse polarization, *i.e*., for *ImF_x_*_(_*_y_*_)_(*k, ω*). This function is exactly zero only for the region for arguments 0 < *kd − ωd/v* < 2*π* and 0 < *kd* + *ωd/v* < 2*π*, where one can write:

(27) ImFx(y)(k,ω)=−∑m=1∞[sin(m(kd+ωd/v))−sin(m(kd−ωd/v))m3−(ωd/v)cos(m(kd+ωd/v))+cos(m(kd−ωd/v))m3−(ωd/v)2sin(m(kd+ωd/v))−sin(m(kd−ωd/v))m3]+23(ωd/v)3=−[π26(kd+ωd/v)−π4(kd+ωd/v)2+112(kd+ωd/v)3]+[π26(kd−ωd/v)−π4(kd−ωd/v)2+112(kd−ωd/v)3]+(ωd/v)[π26−π2(kd+ωd/v)+14(kd+ωd/v)2]+(ωd/v)[π26−π2(kd−ωd/v)+14(kd−ωd/v)2]12(ωd/v)2[π−kd−ωd/v]−12[π−kd+ωd/v]+23(ωd/v)3≡0.

Nevertheless, outside the region 0 < *kd ± ωd/v* < 2*π*, the value of *ImF_x_*_(_*_y_*_)_ is not zero, as is demonstrated in Figure 3, and can be accounted for by the formula (*x* = *kd*, *y* = *d/a*):

(28) $$\begin{array}{l} ImF_{x(y)}(k,\omega )=-\Theta (2\pi -x-\omega ya/v)\left[\frac{\pi^{2}}{6}(x+\omega ya/v)-\frac{\pi }{4}{\left(x+\omega ya/v\right)}^{2}+\frac{1}{12}{\left(x+\omega ya/v\right)}^{3}\right]\\ -\Theta (-2\pi +x+\omega ya/v)\left[\frac{\pi^{2}}{6}(x+\omega ya/v-2\pi )-\frac{\pi }{4}{\left(x+\omega ya/v-2\pi \right)}^{2}+\frac{1}{12}{\left(x+\omega ya/v-2\pi \right)}^{3}\right]\\ +\Theta (x-\omega ya/v)\left[\frac{\pi^{2}}{6}(x-\omega ya/v)-\frac{\pi }{4}{\left(x-\omega ya/v\right)}^{2}+\frac{1}{12}{\left(x-\omega ya/v\right)}^{3}\right]\\ +\Theta (-x+\omega ya/v)\left[\frac{\pi^{2}}{6}(x-\omega ya/v+2\pi )-\frac{\pi }{4}{\left(x-\omega ya/v+2\pi \right)}^{2}+\frac{1}{12}{\left(x-\omega ya/v+2\pi \right)}^{3}\right]\\ +\Theta (2\pi -x-\omega ya/v)(\omega ay/v)\left[\frac{\pi^{2}}{6}-\frac{\pi }{2}(x+\omega ya/v)+\frac{1}{4}{\left(x+\omega ya/v\right)}^{2}\right]\\ +\Theta (x-\omega ay/v)(\omega ay/v)\left[\frac{\pi^{2}}{6}-\frac{\pi }{2}(x-\omega ya/v)+\frac{1}{4}{\left(x-\omega ya/v\right)}^{2}\right]\\ +\Theta (-2\pi +x+\omega ay/v)(\omega ay/v)\left[\frac{\pi^{2}}{6}-\frac{\pi }{2}(x+\omega ya/v-2\pi )+\frac{1}{4}{\left(x+\omega ya/v-2\pi \right)}^{2}\right]\\ +\Theta (-x+\omega ay/v)(\omega ay/v)\left[\frac{\pi^{2}}{6}-\frac{\pi }{2}(x-\omega ya/v+2\pi )+\frac{1}{4}{\left(x-\omega ya/v+2\pi \right)}^{2}\right]\\ +\Theta (2\pi -x-\omega ya/v)\frac{1}{2}{\left(\omega ay/v\right)}^{2}\left[\pi -x-\omega ya/v\right]+\Theta (-2\pi +x+\omega ya/v)\frac{1}{2}{\left(\omega ay/v\right)}^{2}\left[3\pi -x-\omega ya/v\right]\\ -\Theta (x-\omega ya/v)\frac{1}{2}{\left(\omega ay/v\right)}^{2}\left[\pi -x+\omega ya/v\right]-\Theta {\left(-x+\omega ay/v\right)}^{2}\left[-\pi -x+\omega ya/v\right]+\frac{2}{3}{\left(\omega ay/v\right)}^{3}. \end{array}$$

**Figure 4.**
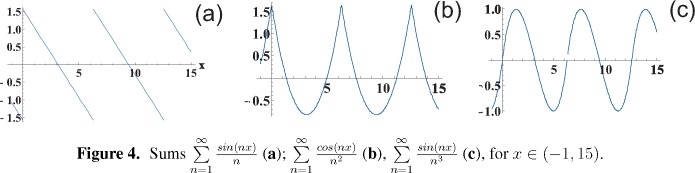
Sums $\sum_{n=1}^{\infty }\frac{sin(nx)}{n}$ (**a**); $\sum_{n=1}^{\infty }\frac{cos(nx)}{n^{2}}$ (**b**), $\sum_{n=1}^{\infty }\frac{sin(nx)}{n^{3}}$ (**c**), for *x* ∈ (−1, 15).

This function is plotted in Figure 3. The discontinuity jump on the border between the regions with the vanishing radiative damping and with the nonzero radiative attenuation is caused by the discontinuous function $\sum_{n=1}^{\infty }\frac{sin(nz)}{n}$ (*cf*. Figure 4) entering *ImF_x_*_(_*_y_*_)_, but not *ImF_z_* (*cf*. Equation (23)).

**Figure 5.**
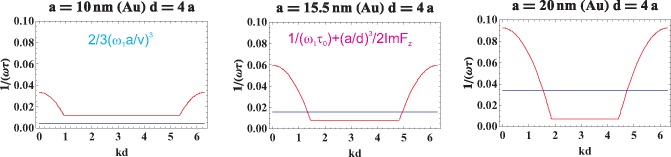
Comparison of various contributions to collective plasmon damping in the chain, for the longitudinal polarization; the red line corresponds to the total damping rate (in dimensionless units) $\frac{1}{\omega_{1}\tau_{z}}=\frac{1}{\omega_{1}\tau_{0}}+\frac{a^{3}}{2d^{3}}ImF_{z}(k)$ and the bottom of this line plot displays only the Ohmic contribution at that segment of the *k* period where all e-mlosses vanish; the blue line indicates the Lorentz friction value (in dimensionless units) $\frac{1}{3}{\left(\frac{\omega_{1}a}{v}\right)}^{3}$.

**Figure 6.**
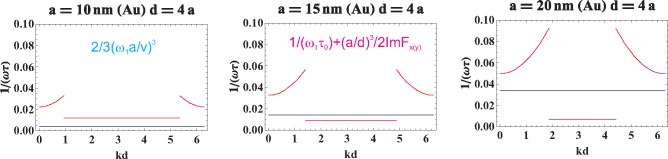
Comparison of various contributions to collective plasmon damping in the chain, for the transverse polarization; the red line corresponds to the total damping rate (in dimensionless units). $\frac{1}{\omega_{1}\tau_{x(y)}}=\frac{1}{\omega_{1}\tau_{0}}+\frac{a^{3}}{2d^{3}}ImF_{x(y)}(k)$, and the bottom of this line plot displays only the Ohmic contribution at that segment of the *k* period where all e-m losses vanish; the blue line indicates the Lorentz friction value (in dimensionless units) $\frac{1}{3}{\left(\frac{\omega_{1}a}{v}\right)}^{3}$.

In order to compare the magnitudes of various contributions to the damping of collective plasmons in the chain, let us plot dimensionless values, for the longitudinal polarization, $\frac{1}{\omega_{1}\tau }=\frac{1}{\omega_{1}\tau_{0}}+\frac{a^{3}}{2d^{3}}ImF_{z}\left(k\right)$ (in red in Figure 5) in comparison to the Lorentz friction contribution $\frac{1}{3}{\left(\frac{\omega_{1}a}{v}\right)}^{3}$ (blue line in Figure 5; *cf*. Equation (30)). In this figure, one can note that for small nanospheres (*a* = 10 nm), the Lorentz term is lower than the Ohmic attenuation (the bottom of the red line; *cf*. also Equation (4)), while for larger nanospheres (*a* = 15, 20 nm), the Lorentz friction dominates as proportional to *a*^3^. For these larger nanospheres, the Ohmic damping is also lower due to *a^−^*^1^ term in Equation (4). The same can be demonstrated for the transverse polarization, as is shown in Figure 6.

## 5. Plasmon-Polariton Self-Modes in the Chain and Their Propagation along Periodic Metallic Nanostructure

The real part of the functions *F*_α_ renormalizes the self-frequency of plasmon-polaritons in the chain, while the imaginary part renormalizes damping of these modes. *ReF_α_*(*k*, ω) and *ImF*_α_(*k*, ω) are functions of *k* and ω. With the first order approximation (the perturbation approach), one can put ω = ω_1_ in *ReF*_α_ and also in the residual nonzero *ImF*_α_, the latter outside the region 0 < *kd* ± ω_1_*d/v* < 2*π*. Let us emphasize, however, that vanishing of *ImF_α_*(*k*, ω) inside the region 0 < *kd* ± ω*d/v* < 2*π* holds for any value of ω [49] (thus also for the exact solution for frequency and not only for ω = ω_1_).

Therefore, one can rewrite the dynamic Equation (19) for plasmon-polariton modes in the chain in the following form:

(29) $$\left(-\omega^{2}-i\frac{2}{\tau_{\alpha }(k)}\omega +\omega_{\alpha }{\left(k\right)}^{2}\right)D_{\alpha }(k,\omega )=\varepsilon a^{3}\omega_{1}^{2}E_{0\alpha }(k,\omega ),$$

where the renormalized attenuation rate (with the perturbation approach),

(30) $$\frac{1}{\tau_{\alpha }(k)}=\left\{ \begin{array}{l} \frac{1}{\tau_{0}},\;for\;0<kd\pm \omega_{1}d/v<2\pi \\ \frac{1}{\tau_{0}}+\frac{a^{3}\omega_{1}}{2d^{3}}ImF_{\alpha }(k,\omega_{1}),\;for\;kd-\omega_{1}d/v<0\;or\;kd+\omega_{1}d/a>2\pi \end{array}\right.$$

and the renormalized self-frequency (with the perturbation approach),

(31) $$\omega_{\alpha }^{2}(k)=\omega_{1}^{2}\left(1-\frac{a^{3}}{d^{3}}ReF_{\alpha }(k,\omega_{1})\right).$$

Equation (29) can be easily solved both for the inhomogeneous and homogeneous (when *E*_0_*_α_* = 0) case. The general solution of Equation (29) has the form of the sum of the general solution of the homogeneous equation and of the single particular solution of the inhomogeneous equation. The first one includes initial conditions and describes damped self-oscillations with the frequency,

(32) $${\omega^{\prime }}_{\alpha }=\sqrt{\omega_{\alpha }^{2}(k)-\frac{1}{\tau_{\alpha }^{2}(k)}},$$

*i.e.*, for each *k* and *α*,

(33) $${D^{\prime }}_{\alpha }(k,t)=A_{\alpha ,k}e^{i{\left({\omega^{\prime }}_{\alpha }t+\phi_{\alpha ,k}\right)}_{e^{-t/\tau_{\alpha }(k)}}},$$

where constants *A_α,k_* and *ϕ_α,k_* are adjusted to initial conditions.

For the inhomogeneous case, the particular solution is as follows:

(34) $${D^{\prime \prime }}_{\alpha }(k,t)=\varepsilon a^{3}\omega_{1}^{2}E_{0\alpha }(k)e^{i(\gamma t+\eta_{\alpha ,k})}\frac{1}{\sqrt{{\left(\omega_{\alpha }^{2}(k)-\gamma^{2}\right)}^{2}+\frac{4\gamma^{2}}{\tau_{\alpha }^{2}(k)}}},$$

suitably for the assumed single Fourier time-component of $E_{0\alpha }(k,t)=E_{0\alpha }(k)e^{i\gamma t}$ and $tg(\eta_{\alpha ,k})=\frac{2\gamma /\tau (\alpha ,k)}{\omega {\left(\alpha ,k\right)}^{2}-\gamma^{2}}$ as usual for a forced oscillator. Let us emphasize that *E*_0_*_α_*(*k*) is the real function for *E*_0_*_α_*(*ld*)^*^ = *E*_0_*_α_*(*ld*) = *E*_0_*_α_*(−*ld*). An appropriate choice of the latter function, in practice a choice of the number of externally-excited nanospheres in the chain, e.g., by a suitably-focused laser beam, allows for modeling of its Fourier picture *E*_0_*_α_*(*k*). This gives the envelope of the wave packet if one inverts the Fourier transform in the solution given by Equation (34) back to the position variable. For the case of the external excitation of only a single nanosphere, the wave packet envelope includes homogeneously all wave vectors *k* ∈ [0, 2*π*). The larger the number of nanospheres that are simultaneously excited, the narrower the envelope for *k* wave packet can be selected. For *E*_0_*_α_*(*ld*)^*^ = *E*_0_*_α_*(*ld*) = *E*_0_*_α_*(−*ld*), the Fourier transform has the same properties, *i.e*., *E*_0_*_α_*(*k*)^*^ = *E*_0_*_α_*(*k*) = *E*_0_*_α_*(−*k*), and the latter equality can be rewritten, due to the period $\frac{2\pi }{d}$ for *k*, as $E_{0\alpha }(-k)=E_{0\alpha }(\frac{2\pi }{d}-k)=E_{0\alpha }(k)$. The inverse Fourier picture of Equation (34) (its real part) has the form,

(35) $${D^{\prime \prime }}_{\alpha }(ld,t)=\int_{0}^{2\pi /d}dkcos(kld-\gamma t-\eta_{\alpha ,k})\varepsilon a^{3}\omega_{1}^{2}E_{0\alpha }(k)\frac{1}{\sqrt{{\left(\omega_{\alpha }^{2}\left(k\right)-\gamma^{2}\right)}^{2}+\frac{4\gamma^{2}}{\tau_{\alpha }^{2}(k)}}}.$$

This integral can be rewritten by virtue of the mean value theorem as follows,

(36) $${D^{\prime \prime }}_{\alpha }(ld,t)=\frac{2\pi }{d}\cos(k*ld-\gamma t-\eta_{\alpha ,k*})\varepsilon a^{3}\omega_{1}^{2}E_{0\alpha }(k*)\frac{1}{\sqrt{{\left(\omega_{\alpha }{\left(k*\right)}^{2}-\gamma^{2}\right)}^{2}+\frac{4\gamma^{2}}{\tau_{\alpha }^{2}(k*)}}}.$$

The above expression describes the undamped wave motion with frequency *γ* and the velocity, amplitude and phase shift determined by k^*^. The amplitude attains its maximal value at the resonance, when

(37) $$\gamma =\omega_{\alpha }(k*)\sqrt{1-\frac{2}{{\left(\tau_{\alpha }(k*)\omega_{\alpha }(k*)\right)}^{2}}}.$$

In the chain being the subject of persistent time-dependent electric field excitation applied to some number (even small number) of nanospheres, one deals with undamped wave packet propagating along the whole chain. These modes depend on the particular shaping of the wave packet by the specific choice of the chain excitation, which may be responsible for the experimentally-observed long-range, practically undamped plasmon-polariton propagation [11,24,27,28].

The self-modes described by Equation (33) are damped, and their propagation depends on appropriately-prepared initial conditions admitting nonzero values of *A_α,k_*. These initial conditions might be prepared by switching off the time-dependent external electric field exciting initially some fragment of the chain. The resulting wave packet may embrace the wave-numbers *k* from some region of [0, 2*π*). If only wave-numbers *k*, for which 0 < *kd ± ω*_1_*d/v* < 2*π*, contribute to the wave packet, its damping is only of the Ohmic-type (as shown in Section 4). The value of corresponding $\frac{1}{\tau_{0}}$ lowers with growing *a* (*cf*. Equation (4)); thus, a longer range of these excitations in the chain can be obtained for larger spheres. The limiting value of $\frac{1}{\tau_{0}}=\frac{v_{f}}{2\lambda_{B}}\sim 10^{13}\;1/s$ gives the maximal range of propagation for these modes of plasmon-polariton ∼ 0.1*cτ*_0_ ∼ 10^−6^ m; for the group velocity of the wave packed, we assumed ∼ 0.1*c*, as its maximum value (though depending on the radius and the separation of nanospheres). The group velocity calculated for both polarizations is presented in Section 6.

Though the presented above analysis is addressed to chains consisting of ideal nanospheres, the conclusions hold also for other shape particle chains and meet the experimental observations, at least qualitatively. In [28], the propagation of plasmon-polaritons in the nano-chain of silver rod-shaped particles (90:30:30 nm oriented with the longer axis perpendicularly to the chain in order to enhance near-field coupling [27], with separation face to face of 70 nm) is evidenced by observing the luminescence of the dye particle located in proximity to the transmitting e-m signal, but distant from the point-like excitation source over the range of 0.5 µm. The observed behavior has been supported by FDTDnumerical simulations. Several samples of the chain were fabricated by electron beam lithography in the form of a 2D matrix with sufficiently well-separated individual chains. The energy blue-shift of plasmon resonance for the nano-rods in the chain in comparison to a single particle is observed as *ca*. 0.1 eV (*cf*. Figure 4 in [28]). This agrees with our estimation of the reducing radiation losses in the chain in comparison to the strong Lorentz friction for a single metallic nano-particle and the related smaller red-shift of damped oscillations. The position of resonance maximum in the chain is located at higher energy for transverse polarization mode than for the longitudinal one [26], which also agrees with the theoretical predictions. In [27] it is indicated that FDTD simulations give lower values of the group velocities for both polarizations and higher attenuation rates in comparison to these quantities previously estimated [25,26] with the simplified point-dipole model with near field-field interaction only and neglecting retardation effects. Let us note, however, that the simplified approach, including only the near-field contribution to the electric field of interacting dipoles, leads to an artifact, *i.e.*, for some values of *d* and *a* chain parameters, the instability of collective dynamics occurs [50]. This instability is completely removed by the inclusion of medium- and far-field contributions to the electric field of the dipole and by rigorous inclusion of the relativistic retardation [49]. Nevertheless, the dipole interaction model, even if including, besides the near-field contribution, also medium- and far-field ones and all retardation effects, still suffers from the absence of the magnetic field component needed for the complete description of far-field wave propagation, which might be of particular significance for ferromagnetic metallic nano-chains. Moreover, as has been demonstrated in [51–53], for large separations in the chain, the scattering of e-m radiation dominates the signal behavior in metallic nano-chains, which then acts as the Bragg grating for plasmon-polaritons. For ellipsoidal gold nanoparticles (210:80 nm) deposited on the top of a silicon wave-guide, the change of regime from collective plasmon-polariton guiding to the Bragg scattering scenario takes place at distances between nanoparticles exceeding *ca*. 1 µm [51,53]. This proves that the model of dipole coupling in the nano-chain works quite well in a wide region of chain parameters, in practice up to micron order for distances between metallic elements in the chain, which supports the qualitative argument that the Bragg grating regime is not efficient for sub-wave-length distances and justifies the applicability of the model considered in the present paper. The SNOMmeasurements of near-field coupled plasmon modes in metallic nano-chains [10] interpreted with the classical field-susceptibilities formalism [52] also support the sufficient level of accuracy of dipole approximation for the interaction in the chain for the here considered scale of nanosphere radii *a* of order of 10–30 nm and the chain separation *d* not exceeding ∼ 10*a*.

## 6. Calculation of the Self-Frequencies and Group Velocities of Plasmon-Polaritons in the Metallic Nano-Chain

According to Equations (31) and (32) for self-frequencies of plasmon-polaritons in the chain, one can write out their explicit form using expressions for *F_α_*(*k, ω*_1_) given by Equation (20). The real part of the functions *F_α_*(*k, ω*_1_) renormalizes the corresponding frequencies for both polarizations, and they attain the following form (still with the perturbative approach, according to Equation (31)):

(38) $$\begin{array}{l} \omega_{z}^{2}(k)=\omega_{1}^{2}\left[1-\frac{a^{3}}{d^{3}}4\sum_{m=1}^{\infty }\left(\frac{\cos(mkd)}{m^{3}}\cos(m\omega_{1}d/v)+\omega_{1}d/v\frac{\cos(mkd)}{m^{2}}\sin(m\omega_{1}d/v)\right)\right]\\ \;=\omega_{1}^{2}\left[1-\frac{a^{3}}{d^{3}}2\sum_{m=1}^{\infty }\left(\frac{\cos(m(x+\omega_{1}ya/v))+\cos(m(x-\omega_{1}ya/v))}{m^{3}}+\omega_{1}ya/v\frac{\sin(m(x+\omega_{1}ya/v))-\sin(m(x-\omega_{1}ya/v))}{m^{2}}\right)\right], \end{array}$$

(39) $$\begin{array}{l} \omega_{x(y)}^{2}(k)=\omega_{1}^{2}[1+\frac{a^{3}}{d^{3}}2\sum_{m=1}^{\infty }(\frac{\cos(mkd)}{m^{3}}\cos(m\omega_{1}d/v)+\omega_{1}d/v\frac{\cos(mkd)}{m^{2}}\sin(m\omega_{1}d/v)\\ \;-{\left(\omega_{1}d/v\right)}^{2}\frac{\cos(mkd)}{m}\cos(m\omega_{1}d/v))]\\ \;=\omega_{1}^{2}[1+\frac{a^{3}}{d^{3}}\sum_{m=1}^{\infty }(\frac{\cos(m(x+\omega_{1}ya/v)+\cos(m(x-\omega_{1}ya/v))}{m^{3}}+\omega_{1}ay/v\frac{\sin(m(x+\omega_{1}ya/v))-\sin(m(x-\omega_{1}ya/v))}{m^{2}}\\ \;-{\left(\omega_{1}ay/v\right)}^{2}\frac{\cos(m(x+\omega_{1}ya/v))+\cos(m(x-\omega_{1}ya/v))}{m})], \end{array}$$

where *x* = *kd* and *y* = *d/a*.

**Figure 7.**
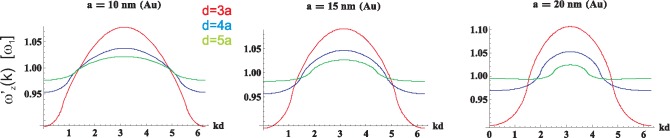
The self-frequency ${\omega^{\prime }}_{z}(k)$ of the plasmon polariton with longitudinal polarization in infinite chains of Au nanospheres with radius *a* = 10, 15, 20 nm and chain-separation *d* = 3*a*, 4*a*, 5*a* (with the perturbation approach).

**Figure 8.**
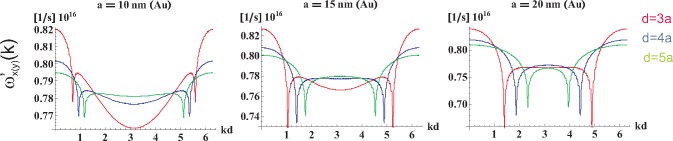
The self-frequency ${\omega^{\prime }}_{x(y)}(k)$ of the plasmon polariton with transverse polarization in infinite chains of Au nanospheres with radius *a* = 10, 15, 20 nm and chain-separation *d* = 3*a*, 4*a*, 5*a* (with the perturbation approach). The logarithmic singularities are the artifact of the perturbation method of the solution of Equation (19); in the exact solution of this equation, all singularities are quenched (*cf*. Section 7).

The shift of self-frequencies of plasmon-polaritons caused by their attenuation is accounted for by Equation (32) where *ω_α_*(*k*) is given by Equations (38) and (39) where *τ_α_*(*k*) has the form as in Equation (30). Taking into account the explicit form for *ImF_α_*(*k, ω*_1_), *i.e.*, Expressions (26) and (28), one can easily calculate the self-frequencies ${\omega^{\prime }}_{\alpha }(k)$; these functions are shown in Figures 7 and 8 for longitudinal and transverse polarizations, respectively.

**Figure 9.**
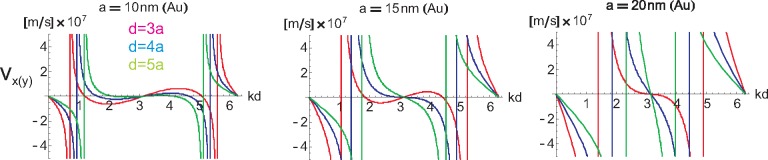
The group velocity *v_x_*_(_*_y_*_)_(*k*) of the plasmon polariton with the transverse polarization in infinite chains of Au nanospheres with radius *a* = 10, 15, 20 nm and chain-separation *d* = 3*a*, 4*a*, 5*a* calculated with the perturbation approach; the hyperbolic singularity is an artifact of the perturbation method of the solution of Equation (19); the exact solution of this equation reduces this singularity to a narrow local jump of the group velocity not exceeding *c* (*cf*. paragraph 7); the small asymmetry of approximated singularities is caused by the imposition of the hyperbolic singularity with the additional logarithmic singularity of *v_x_*_(_*_y_*_)_(*k*) in the same points.

Note that for the transverse polarization in Equation (39) the sum,

(40) $$\sum_{m=1}^{\infty }\frac{\cos(m(x+\omega_{1}ya/v))+\cos(m(x-\omega_{1}ya/v))}{m},$$

can be performed analytically according to Equation (24) resulting in the contribution,

(41) $$-\frac{1}{2}\ln[(2-2\cos(x+\omega_{1}ya/v))(2-2\cos(x-\omega_{1}ya/v))]$$

(the other sums in Equations (38) and (39) have to be done numerically). This logarithmic singularity in self-frequencies for transverse plasmon-polaritons on the rim of the region 0 < *x* − *ω*_1_*ya/v* < 2*π* (inside which radiative damping vanishes) is indicated in Figure 8. This singularity causes hyperbolic discontinuity in transverse polarization mode group velocity (*cf*. Figure 9). Nevertheless, the logarithmic singularity in the self-energy and the related hyperbolic discontinuity in the group velocity for the transverse polarization turn out to be artifacts of the perturbation solution of Equation (19). The exact numerical solution of this equation demonstrates the effective quenching of the logarithmic singularity to the small local minimum, resulting in the finite group velocity discontinuity, as shown in Section 7. This property of the transverse polarization mode of plasmon-polaritons in the chain caused by the interference of the far-field component of dipole interactions between nanospheres was analyzed numerically in [32,54] and commented on in [49,55]. The numerical studies of plasmon-polariton propagation in metallic nano-chains [32–34,54] indicated a very narrow and weak, but long-range mode, besides the wide spectrum of quickly damped modes. This ‘fainting’long-range mode has been associated with the constructive interference of the far-field part of the dipole-dipole interaction of nanospheres in the chain, resulting in local enhancement of the transverse polarization mode group velocity in the narrow vicinity of singularity points (on the light cone).

**Figure 10.**
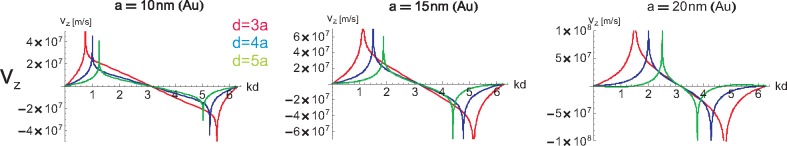
The group velocity *v_z_*(*k*) of the plasmon polariton with the longitudinal polarization in infinite chains of Au nanospheres with radius *a* = 10, 15, 20 nm and chain-separation *d* = 3*a*, 4*a*, 5*a* calculated with the perturbation approach; the logarithmic singularity in the perturbation formula for *v_z_* (though not the formula for ${\omega^{\prime }}_{z}$) leads to the local exceeding of *c*; this artifact of the perturbation approach is removed by the exact solution (*cf*. Section 7).

In order to find the group velocities of particular self-modes of plasmon-polaritons in the chain, the derivative of ${\omega^{\prime }}_{\alpha }(k)$ with respect to *k* must be performed, which according to Equations (30), (32), (38) and (39) is straightforward though extended calculus. The sums in the formulae for *ω_α_*(*k*) still cannot be done analytically, except for the sum with denominator *m* in Equation (39). The resulting group velocity calculated numerically for both polarizations and for *kd* ∈ [0, 2*π*) and *d/d* ∈ [3, 10] are presented in Figures 9 and 10 (for *a* = 10, 15, 20 nm for both polarizations).

## 7. Exact Solution of Equation (19): The Resolution of the Problem of the Logarithmic Divergence of the Far-Field Contribution to the Self-Frequency for the Transverse Polarization of Plasmon Polaritons and of the Medium-Field Contribution to Group Velocities for Both Polarizations

**Figure 11.**
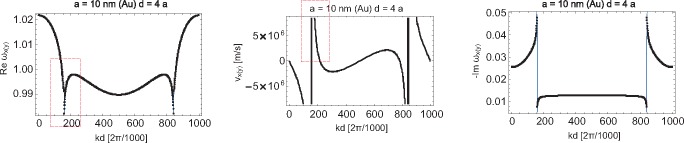
The exact solution for the self-frequency and the damping rate of the transverse polarization mode of plasmon-polaritons in the nano-chain (*ω* in *ω*_1_ units; solution of Equation (19) in 1000 points on the segment [0,2*π*); in the middle, the plot for the group velocity of the transverse polarization mode is presented with hyperbolic-type singularities corresponding to logarithmic-type singularities of self-energy (left) mixed with the additional logarithmic-type singularity of the group velocity itself; all singularities are, however, truncated, which is visualized in the marked regions in Figure 12.

**Figure 12.**
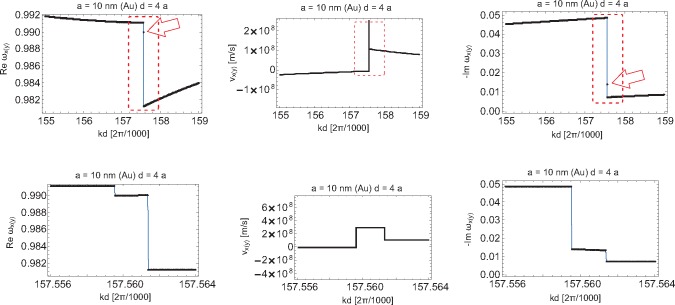
Two-level magnification of the scale view of the truncated singularity region for the transverse plasmon-polariton mode in the nano-chain for the exact solution of Equation (19); the most enhanced scale is in the bottom panels (*ω* in *ω*_1_ units).

**Figure 13.**
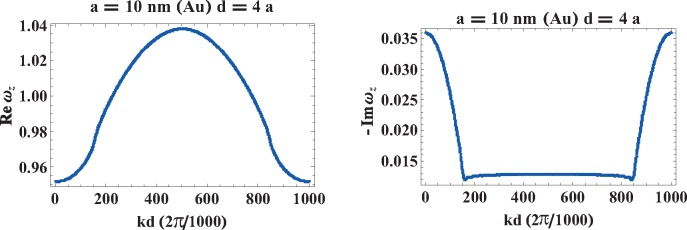
The exact solution for the self-frequency and the damping of the longitudinal mode of plasmon-polaritons in the nano-chain (*ω* in *ω*_1_ units).

**Figure 14.**
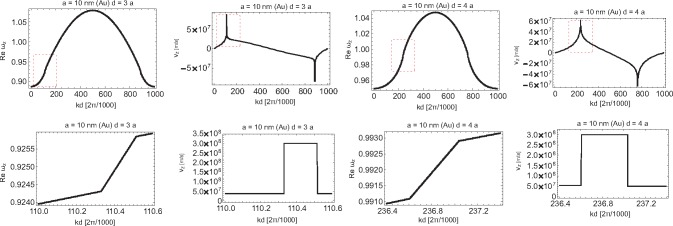
The exact solution for the group velocity *v_z_* of the longitudinal mode of plasmon-polaritons in the nano-chain for *a* = 10 nm and *d* = 3*a*, 4*a*, respectively, and the corresponding exact solutions for *Re*ω*_z_* (left); the exact solution of the dynamic Equation (19) removes the logarithmic singularity in the group velocity; the remaining local very narrow extremes are truncated exactly at *c* (in close vicinity to the singular points, the solution was found in more than 1000 points *kd*; in the bottom panels, plots are visualized corresponding to the marked fragments in the upper panels in the enhanced scale).

The imaginary part of the complex ω, the solution of Equation (19), defines plasmon-polariton attenuation, while the real part of ω gives the self-frequency of these oscillations (in the case of the homogeneous equation, *i.e.*, when *E*_0_*_α_*(*k*, ω) = 0). The derivative of this self-frequency with respect to the wave vector *k* defines the group velocity of particular modes. Because of the logarithmic singular term in the far-field transverse contribution to the dipole interaction in the chain,

(42) $$\begin{array}{l} \sum_{m=1}^{\infty }\frac{\cos(m(x+\omega_{1}ya/v))+\cos(m(x-\omega_{1}ya/v))}{m}\\ =-\frac{1}{2}\ln[(2-2\cos(x+\omega ya/v))(2-2\cos(x-\omega ya/v))], \end{array}$$

one cannot apply the perturbation method for the solution of the dynamical equation, at least in the region close to the singularity (on the light cone). Note that with the perturbation approach, one substitutes ω with ω_1_ (Mie frequency) in the r.h.s. of Equation (19). This produces, however, the hyperbolic singularity in transverse group velocity by virtue of Equation (42). Moreover, with the perturbation approach, the logarithmic singularity occurs for both polarizations, which is noticeable if one takes the derivative with respect to *k* from Equations (38) and (39). All of these singularities occur at isolated points for which *kd* ± ω_1_*d/v* = *l*π (where *l* is an integer) integer). Both hyperbolic and logarithmic divergences for the perturbation formula for group velocities at this point would result in locally exceeding *c* by corresponding group velocities. To resolve the problem of this unphysical divergence, the exact solution of Equation (19) must be found; because of the divergence of Equation (42), the corresponding contribution cannot be treated any longer as the perturbation. The exact solution of Equation (19), found numerically by use of the Newton-type procedure in 1000 points for *kd* ∈ [0, 2*π*) [55], is plotted in Figures 11–14, for both polarizations of plasmon-polaritons.

From Figures 11 and 13, we notice by comparison with the corresponding plots obtained with the perturbation method that for the longitudinal polarization, the exact solutions for self-frequencies do not differ significantly from those obtained in the perturbation manner, but the change suffices to remove the logarithmic divergence from the derivative of the self-frequency. For the transverse polarization, the difference is also not important for the attenuation plot. However, for the transverse polarization self-frequency in the case of the exact solution, we deal with quenching of the logarithmic divergence (42), contrary to its approximated version (obtained with the perturbation approach). Instead of the infinite singularity, we observe in the exact plot for the transverse polarization self-frequencies only the relatively small minimum, resulting then conveniently in finite group velocity no greater than *c*. This quenched logarithmic singularity into the small local minimum is presented in Figure 12 (right; on the left, the correction to the discontinuity step in the damping of the transverse mode caused by the logarithmic contribution to Equation (19) is also presented).

The exact solution of the dynamic Equation (19) resolves thusly the problem of the risky logarithmic divergent contribution of the transverse far-field part of the dipole interaction of nanospheres in the infinite chain and regularizes the final solution for corresponding characteristics of plasmon-polariton modes [55]. In the vicinity of singularity points (in the domain for *kd*), the group velocity, though still well below the light velocity, is, however, greater in comparison to group velocities in other regions of the *k* wave vector domain. This might elucidate the former numerical observations [54] of the long-range propagating mode for the transverse polarization of the plasmon-polariton in the nano-chain. In view of the above analysis, one can can argue that the long range of the related mode [54] is connected to a locally higher value of its group velocity, but not to lowering of its damping.

Even though the real part of the function *F_z_*, and thus, ω*_z_*(*k*) (Equation (38)), is given by a continuous function, the corresponding group velocity will have a logarithmic singularity, as the derivative of ω*_z_*(*k*) with respect to *k* will contain the sum (cos(*m*(*x* + ω_1_*ya/v*)) − *cos*(*m*(*x* − ω_1_*ya/v*)))*/m*, arising from the last term in Equation (38) after taking the derivative. A similar term is present also in the perturbation formula for ω*_x_*_(_*_y_*_)_(*k*) in Equation (39). The origin of these terms for both polarizations is the medium-field contribution to the dipole interaction in the chain. In points *x* ± ω_1_*ya/v* = *p*2*π*, *p* integer, logarithmic singularity produces an artifact of the group velocity *v_z_* exceeding *c*. This precludes the applicability of the perturbation solution approach, at least close to singularity points. Therefore, instead of putting ω = ω_1_ in function *F_α_* in the r.h.s. of Equation (16) (as was done for the perturbation method of the solution of this equation), one must solve the nonlinear equation (in the homogeneous case neglecting *E*_0_*_α_*). As was already mentioned above, this exact solution can be found numerically by a Newton-type procedure, and both real and imaginary parts of ω can be determined, point by point, in the whole region *kd* ∈ [0, 2*π*). For the longitudinal polarization of the group velocity, the exact solution is presented in Figure 14. The exact solutions for *v_z_* do not exhibit any singularities: the logarithmic singularity of the perturbation term is quenched to only small local extrema, similar to what was demonstrated above for the transverse polarization.

The logarithmic-type singularity in the self-energy of transverse plasmon-polaritons in the chain is the feature that essentially differentiates these modes from the longitudinally-polarized ones. This singularity is caused by the sum of far-field pieces of the electric field of all nanoparticle dipoles, which influences charge oscillations in each component of the chain and produces the hyperbolic-type discontinuity in group velocity exclusively for the transverse polarization. Besides this discontinuity, the medium-field component of the electric interaction of dipoles additionally produces a logarithmic-type singularity in the group velocity for both polarizations (though any singularity in the self-energy). We use here the terms logarithmic-type or hyperbolic-type singularities to distinguish the exact behavior of the group velocities obtained by the exact solution for self-energies for both polarizations, which are sharpened and truncated at *c* due to relativistic constraints imposed on the dynamic equation and manifesting themselves in the form of its solution. The retardation of electric signals prohibits the collective excitation group velocity from exceeding the light velocity. This quenching concerns infinite singularities, which occur in perturbation expressions for self-energy and next in perturbation formulae for group velocities. The relativistic invariance of the dynamic equation for collective dipole plasmon oscillations in the chain prevents, however, the group velocity of particular plasmon-polariton modes from exceeding the light velocity. Thus, the exact solution of this equation inherently posses also this property. Exact self-energies have suitably regularized their dependence with respect to *k*, such that their derivatives do not exceed *c*.

It is worth emphasizing, for the sake of completeness of the description, that inclusion of the magnetic field of dipoles does not modify this scenario, because the magnetic field contribution to self-energies is at least two orders lower in comparison to the electric field contribution due to the Fermi velocity of electrons being two orders lower in comparison to the light velocity, which significantly reduces the Lorentz force. Therefore, the magnetic field of the dipoles [39,47],

(43) $$B_{\omega }=ik(D_{\omega }\times n)\left(\frac{ik}{r_{0}}-\frac{1}{r_{0}^{2}}\right)e^{ikr_{0}},$$

though contributing to the far-field and medium-field parts of plasmon-polariton self-energies, does not significantly change the similar terms caused by the electric field and causes, only by two-orders, lower corrections, which are practically negligible.

It must be, however, emphasized that the role of the magnetic field may change considerably in the case of magnetic-type metallic chains. For a ferromagnetic material used to prepare chain components, one can expect a strengthening of the magnetic field effects in surface plasmon collective dynamics in the chain. Thus, the consideration presented in the paper, while appropriate for noble metals, ought to be lifted to much more complicated magneto-plasmonics for magnetic metals. This is, however, beyond the scope of the present paper.

The distinction between truncated singularities in group velocities for transverse and longitudinal polarizations of plasmon-polaritons in the chain as demonstrated above sheds light on recent the discussion of long-range plasmon-polariton modes in metallic nano-chains studied by numerical simulations with the Green-function method for differential equations [32,33,35]. The truncated singularities in group velocities indicated above, *i.e.*, the very narrow logarithmic-type truncated singularity for the longitudinal polarization and the wider hyperbolic-type one mixed also with a narrow logarithmic-type truncated singularity for the transverse polarization, give rise to understanding the peculiarities of numerical studies. The long-range propagation of narrow modes observed in numerical simulations might be linked to the local enhancement of the group velocity of *k*-modes in singular regions. In the simulations [32,33,54], it was assumed that the single selected nanosphere in the chain was initially excited, and then, the range of propagation for various modes of plasmon-polariton was observed. The point-like initial excitation corresponds in the Fourier picture to uniform excitations of all *k* modes, including also those in singular regions. The different character of the truncated singularities, originally being infinite divergences in the perturbation series, for the longitudinal and the transverse polarization will result in the different features of the corresponding modes (the local narrowing of the group velocity curve is stronger for the longitudinal polarization). Even though the group velocity singularities are truncated on the finite *v* = *c* level in a different fashion from the transverse and the longitudinal polarization, the local narrow extrema give the same long range for the propagation of closely located modes for both polarizations (*ca*. two orders longer than for nonsingular modes).

## 8. Conclusions

In summary, one can state that in infinite metallic nano-chains, the long-range propagation of plasmon-polariton self-modes can be observed due to effective ideal compensation of the Lorentz friction losses, in particular nanospheres by the energy income from other nanospheres in the chain, which takes place for both polarizations of the collective plasmon modes in a relatively large part of the wave vector *k* domain (diminishing with *d/a* growth). In the case of the inhomogeneous equation version of Equation(19) (*i.e.*, when the term of persistent external force is added to its r.h.s.: $\varepsilon a^{3}\omega_{1}^{2}E_{0\alpha }(k,\omega )$, where *ε* is the dielectric susceptibility of the chain surroundings and *E*_0_*_α_*(*k*, ω) is the external electric field Fourier component), one can expect the completely undamped propagation of induced plasmon-polaritons corresponding to the forced oscillator properties. Both the low (only Ohmic) attenuation of self-oscillations and the possibility of exciting appropriately formed (by persistent excitation of some part of the chain) wave packets of plasmon-polaritons modes in the chain may be responsible for the experimentally-observed, practically undamped propagation of the collective plasmon signal over relatively large distances in several µm-length nano-chains.

It must be emphasized that the almost undamped propagation of surface plasmon-polaritons along metallic nano-chains was first analyzed by Markel and developed next by Citrin and, more recently, by other authors. All of them tried to solve the problem of plasmon-polariton propagation with the dipole model of particle interaction. This makes the issue relatively simple and clearly formulated, *i.e*., the corresponding dynamic equation is the linear differential equation, and it can be solved using various techniques. Nevertheless, some methods might not be transparent and additionally might complicate the solution and interpretation. In the present paper, we have solved the linear differential equation by the most standard technique of the Fourier transform (continuous transform (CFT) in time domain and discrete (DFT) in the position domain, as is apparently linked with the problem definition for the periodic chain). All of the analysis is completely and transparently presented mainly in an analytical manner. This is the basic value of this approach for the the wave representation close to the experimental interpretation. The other approaches (e.g., the Z transformation method with the Green function method) give similar results. Unfortunately, none of the applied methods allow for the full analytical solution, and in each approach, some numerical procedure had been applied. As is common in differential equation theory, some small uncertainties in a singular region could cause some misleading conclusions. The effective numerical solution for dispersion may thus have some inherent approximation character connected to a necessary procedure if one cuts the infinite series in the numerical treatment. The solution of the Fourier transform of the initial differential equation is transparent in this regard and exhibits some singular points (corresponding to the constructive interference for transverse polarization of plasmon-polaritons due to the far-field radiation on the light cone). This singularity is visible in all formulations because of the apparent logarithmic divergence of the infinite sum of $\frac{\cos(nx)}{n}$. Various conclusions related to this singularity may be, however, formulated; but not all are correct, especially if formulated with perturbative calculus, as directly demonstrated in the present paper. The only way to resolve this problem is to exactly solve the underlying equation. The exact solution means here the solution of the nonlinear equation with respect to the Fourier argument algebraic equation point-by-point. For singularity points, all methods agree, even approximated ones. Special attention must be given, however, to the group velocity, which in the framework of the above presented Fourier transform can be explicitly expressed and studied revealing the next sources of singularities (hyperbolic for transverse polarization (caused by far-field zone dipole radiation) and the additional logarithmic one (caused by medium-field zone radiation for both transverse and longitudinal polarizations)). These singularities are hard notice in numerical experiments, possibly due to the slight confusion caused by the application of unavoidable approximation methods of numerical calculus. Resolving what the artifact is thus is unclear and might cause doubt. The aim of our paper is to demonstrate directly the character of the above-mentioned singularities in a transparent and highly analytical manner. The singularities in the solution of the differential equation are explicitly demonstrated as being caused by the perturbation methods. Moreover, the exact solution of the problem is described, free of all nonphysical singularities. It might be emphasized that such a level of transparency is not achieved with other techniques where the problem of singularities is not solved in a final manner. Therefore, the complete and transparent solution of the same initial problem is the novel and important contribution to the debate on plasmon-polaritons in metallic nano-structures.

## Figures and Tables

**Table 1.** Radius of the nanosphere corresponding to the minimal value of surface plasmon damping.

$n=\sqrt{\varepsilon }$	a^*^		
	Au	Ag	Cu
*n* = 1 vacuum	8.78	8.77	8.82
*n* = 1.4 water	10.4	10.42	10.5
*n* = 2	12.4	12.41	12.5

## Notes

**PACS classifications**: 36.40.Gk, 73.20.Mf
